# Correction: Enhanced up-conversion luminescence in transparent glass-ceramic containing KEr_3_F_10_:Er^3+^ nanocrystals and its application in temperature detection

**DOI:** 10.1039/c9ra90032b

**Published:** 2019-05-16

**Authors:** Zhijun Xia, Huixiang Huang, Zhi Chen, Zaijin Fang, Jianrong Qiu

**Affiliations:** State Key Laboratory of Luminescent Materials and Devices, Institute of Optical Communication Materials, South China University of Technology Wushan Road 381 Guangzhou 510641 China qjr@scut.edu.cn; State Key Laboratory of Modern Optical Instrumentation, College of Optical Science and Engineering, Zhejiang University Hangzhou 310027 China; Guangdong Provincial Key Laboratory of Optical Fiber Sensing and Communications, Institute of Photonics Technology, Jinan University Guangzhou 510632 China

## Abstract

Correction for ‘Enhanced up-conversion luminescence in transparent glass-ceramic containing KEr_3_F_10_:Er^3+^ nanocrystals and its application in temperature detection’ by Zhijun Xia *et al.*, *RSC Adv.*, 2019, **9**, 10999–11004.

The authors regret that the reported composition of precursor glass in the experimental section of the original article was incorrect. The text should read “The precursor glass (PG) compositions (mol%) is 17.5 KF-17.5 ZnF_2_-65 SiO_2_”.

The Royal Society of Chemistry apologises for these errors and any consequent inconvenience to authors and readers.

## Supplementary Material

